# Controllable preparation of chitosan oligosaccharides *via* a recombinant chitosanase from marine *Streptomyces lydicus* S1 and its potential application on preservation of pre-packaged tofu

**DOI:** 10.3389/fmicb.2022.1007201

**Published:** 2022-09-26

**Authors:** Hao Chen, Bilian Lin, Rui Zhang, Zhouliang Gong, Ming Wen, Weiming Su, Jinsong Zhou, Liangzhong Zhao, Jianrong Wang

**Affiliations:** ^1^College of Food and Chemical Engineering, Shaoyang University, Shaoyang, China; ^2^Hunan Provincial Key Laboratory of Soybean Products Processing and Safety Control, Shaoyang, China; ^3^College of Food Science and Technology, Guangdong Ocean University, Zhanjiang, China; ^4^Guangdong Provincial Key Laboratory of Aquatic Product Processing and Safety, Zhanjiang, China; ^5^Jinzai Food Group Co., Ltd., Yueyang, China; ^6^Shenzhen Raink Ecology and Environment Co., Ltd., Shenzhen, China

**Keywords:** chitosanase, *Streptomyces lydicus* S1, chitosan oligosaccharides, controllable preparation, preservation of tofu

## Abstract

Chitosan oligosaccharides (COSs) are widely applied in many areas due to its various biological activities. Controllable preparation of COSs with desired degree of polymerization (DP) *via* suitable chitosanase is of great value. Herein, a novel glycoside hydrolase (GH) family 46 chitosanase (SlCsn46) from marine *Streptomyces lydicus* S1 was prepared, characterized and used to controllably produce COSs with different DP. The specific activity of purified recombinant SlCsn46 was 1,008.5 U/mg. The optimal temperature and pH of purified SlCsn46 were 50°C and 6.0, respectively. Metal ions Mn^2+^ could improve the stability of SlCsn46. Additionally, SlCsn46 can efficiently hydrolyze 2% and 4% colloidal chitosan to prepare COSs with DP 2–4, 2–5, and 2–6 by adjusting the amount of SlCsn46 added. Moreover, COSs with DP 2–4, 2–5, and 2–6 exhibited potential application value for prolonging the shelf-life of pre-packaged Tofu. The water-holding capacity (WHC), sensorial properties, total viable count (TVC), pH and total volatile base nitrogen (TVB-N) of pre-packed tofu incorporated with 4 mg/mL COSs with DP 2–4, 2–5, and 2–6 were better than those of the control during 15 days of storage at 10°C. Thus, the controllable hydrolysis strategy provides an effective method to prepare COSs with desired DP and its potential application on preservation of pre-packed tofu.

## Introduction

As the derivative products from chitin or chitosan, chitosan oligosaccharides (COSs) are linear co-oligomers which are composed of D-glucosamine (GlcN) and *N*-acetyl-D-glucosamine (GlcNAc) (Kaczmarek et al., 2019). Compared with chitin or chitosan, COSs display several advantages such as high water solubility, low viscosity, biocompatibility as well as biodegradability (Manivasagan et al., 2017; Yuan et al., 2019). As reported, COSs have even better biological activities than chitin or chitosan, including anti-cancer, anti-microbial activity, radical scavenging effects and antioxidant activity, and positive effects on host gut health (Manivasagan et al., 2018; Zhang et al., 2018; Asadpoor et al., 2021; Hao et al., 2021). Based on this, COSs have been widely used in food, biomedical, agriculture, and other fields (Jang et al., 2017; Moorthy et al., 2019). It is particularly noteworthy that the degrees of polymerization (DP) play a pivotal role on the biological activities of COSs (Zhang et al., 2017; Zhao et al., 2022). Some previous studies demonstrated that the COSs with low DP show better antioxidant and anti-inflammatory activities (Liang et al., 2016). On the contrary, several studies found that COSs with high DP are more effective. For instance, Zhang et al. (2017) reported that the growth and photosynthesis of wheat seedlings are closely related to the DP of COSs and DP above three was necessary to insure a significant promotion effect. Additionally, Qian et al. (2016) found that the inhibition activity of chitohexaose against *Escherichia Coli* is about 5-fold higher than that of chitobiose when the concentration is 2.5 mg/mL. Therefore, it is necessary to prepare COSs with desirable DP according to its subsequent industrial application.

Nowadays, the methods for preparation of COSs are mainly composed of chemical, physical, and enzymatic hydrolysis. Among those methods, enzymatic hydrolysis gained great attention due to its advantages such as milder conditions, environmental friendliness as well as DP and process can be controlled (Liaqat and Eltem, 2018; Yuan et al., 2019). According to the results of previous researches, we found that enzymatic method is generally involved with specific and non-specific enzymes (Kaczmarek et al., 2019). As a specific enzyme for preparation of COSs, chitosanase (EC. 3.2.1.132) exhibits its great potential value for industry application. To date, many chitosanases from different sources have been isolated, purified, heterologously expressed, and characterized (Thadathil and Velappan, 2014; Xu et al., 2022). The chitosanases from bacteria and fungi show more competitive than that from plant and cyanobacterial due to their extracellular secretion and excellent biochemical properties (Thadathil and Velappan, 2014). As a kind of glycoside hydrolase (GH), chitosanase is mainly classified into five GH families, including GH5, GH8, GH46, GH75, and GH80. Compared with above mentioned GH families, chitosanases from GH46 families are more suitable for preparation of COSs due to their high efficiency and controllability (Viens et al., 2015; Zhou et al., 2020; Cui et al., 2021). It has been reported that the reaction time for the chitosanase CsnBm from *Bacillus mojavensis* SY1 decomposes 92.3% of 4% colloidal chitosan into COSs with DP from 2 to 6 is only 15 min (Wang et al., 2021a). Similarly, 91.2% of 4% colloidal chitosan could be hydrolyzed and converted into COSs at 20 min by a GH46 chitosanase from *Streptomyces hygroscopicus* R1 (Wang et al., 2021b). According to the previous studies, the chitosanases from GH46 are mainly from bacteria. Among different bacteria, the genus *Streptomyces* is an important producer for preparation of different chitosanases, some of which have been characterized such as ShCsn46, SaCsn46A, Sn1-CSN, CsnN174, and Csn21c from *S. hygroscopicus* R1, *Streptomyces avermitilis*, *Streptomyces niveus*, *Streptomyces sp.* N174, and *Streptomyces albolongus* ATCC 27414, respectively (Guo et al., 2019, 2021; Ding et al., 2020; Chen et al., 2021; Wang et al., 2021b). However, there are few reports about the bioinformatics analysis, heterologous expression, and characterization of chitosanase from *Streptomyces lydicus*.

Tofu, also called soybean curd, has long been preferred by consumers in Asia, especially China, being a delicious and nutritive traditional food. Today, the global consumption of tofu has been increasing, with a growing awareness of the health benefits of soy-based foods as cholesterol-free and low in saturated fat (Lu et al., 2022). However, tofu is easily perishable owing to its rich protein, high moisture content and near-neutral pH value, with a maximum preservation period of 3–4 days even under commercial refrigerated storage (usually 0–10°C) (Chen et al., 2014; Yang X. et al., 2020). Some previous studies have been performed to develop different approaches for the shelf-life extension of tofu. Heat sterilization (pasteurization or microwave treatment) combined with refrigerated storage is widely practiced in pre-packaged tofu storage (Yang X. et al., 2020). Additionally, bactericidal chemical additives or natural plants and animals extracts with antibacterial activities, such as chitosan, curcumin microcapsules, and *premna microphylla turcz* leaves extracts, have been added to pre-packaged tofu as preservatives (No et al., 2007; Wang et al., 2012; Huang et al., 2020). Other emerging techniques, included biopreservative microorganisms (such as bacteriocinogenic *Weissella hellenica* D1501) and various edible coating, also have been tried to delay the spoilage of tofu (Chen et al., 2014; Rahmawati et al., 2017). Although these methods could improve the shelf-life and safety of tofu in different extents, they would remarkably alter sensory characteristics and add the cost. Thus, few of them have been used in the industrial production of pre-packaged tofu. COSs are considered as a promising natural antibacterial agent and has been widely applied in food industry. Singh et al. (2019) reported that 1 g/100 g of COSs was incorporated to inhibit the deterioration of sardine surimi gels during storage at 4°C. Jia et al. (2018) also found that the addition of 1% (w/v) COSs to silver carp inhibited growth of microorganisms and reduced off-flavors. In addition, COSs combined with high voltage cold atmospheric plasma (90% Ar/10% O_2_) extended the shelf life of Asian sea bass slices by at least 12 days (Singh and Benjakul, 2020). Under oxygen-based modified atmospheric packaging condition, adding 400 ppm COSs could prolong the preservation period of tuna slices for 9 days (Singh et al., 2021a). But, no information has been available on the application of COSs in pre-packaged tofu preservation.

In this study, the gene encodes a chitosanase (named SlCsn46) from GH46 family was cloned, bioinformatics analyzed and heterologously expressed. Meanwhile, the recombinant SlCsn46 was purified and characterized. Furthermore, controllable preparation of COSs with different DP by purified SlCsn46 was performed. Finally, the potential application of COSs on preservation of tofu was investigated. The results of this study will provide an efficient chitosanase for controllable preparation of COSs and give some clues on the potential application of COSs on preservation of pre-packaged tofu.

## Materials and methods

### Material and chemicals

The PrimeSTAR^®^ HS (Premix), Ex Taq^®^ DNA polymerase and T4 ligase were purchased from Takara Biotechnology (Dalian, China). Substrates, such as chitin, xylan, microcrystalline cellulose, soluble starch, and powdery chitosan with 85%, 90% and 95% degrees of deacetylation (DA), were from Yuanye Biotechnology (Shanghai, China). Chitosan oligosaccharides with different degree of polymerization (DP 1–6) were purchased from Qingdao BZ Oligo Biotech (Qingdao, China). Non-GMO soybeans (protein content 38%) were originally produced in Canada and supplied by Jinzai Food Group Co., Ltd. (Yueyang, China). All other chemical reagents were of analytical grade and purchased from the Guangzhou Chemical Reagent Co., Ltd. (Guangzhou, China).

### Strains, vector, and medium

The *S. lydicus* S1 was isolated from shrimp shell waste, identified by 16sRNA sequencing and stored at –60°C. The *E. coli* Dh5α and competent cell BL21 (DE3) were purchase from Tiangen Biotech (Beijing, China). The plasmids pMD20T and pET-22b were purchased from Takara Biotechnology (Dalian, China) and General Biotechnology (Chuzhou, China), respectively. Media for *E. coli* was Luria-Bertani with ampicillin (LBA) (LB with 25 μg/mL ampicillin).

### Gene cloning, expression, and purification

The genomic DNA of *S. lydicus* S1 was extracted for gene cloning of *slcsn46*. According to the hypothetical chitosanase sequences from *S. lydicus* A02 (CP007699.2, 1954690–1955571), *S. lydicus* 103 (CP017157.1, 5777610–5778485), and *S. lydicus* M01 (CP086217.1, 6802711–6803598), one pair of primers [*sl*-fw, 5′-ATGCGCACCCGATCGA(C/T)A-3′ and *sl*-rev, 5′-TCAGCCGATGTGG (A/T) AGCT-3′] was designed and used to PCR amplification of *slcsn46*. The obtained PCR product was ligated to pMD20T and transformed to *E. coli* competent cell. The transformants were plated on LBA plates. The positive colonies were checked by colony PCR and DNA sequencing. BLASTn and BLASTx were used to analysis the identity of *slcsn46* and SlCsn46 against to chitosanases from different sources. The prediction of signal peptide and tertiary structure of SlCsn46 were performed by SignalP 5.0 server and SWISSMODEL, respectively. Molecular docking of SlCsn46 with chitohexaose was performed by Autodock vina.

The fragment named *slcsn46s* without signal peptide was obtained by PCR amplification using pMD20T-*slcsn46* as template, *slcsn46*-fw (TAATTCGGATCCGGAAGACCGGGCC CCCGCCCGC), and *slcsn46-*rev (TGGTGCTCGAGGCCGAT GTGGAAGCTCTCCCC) as primers. Then, *slcsn46s* was ligated to pET-22b to form expression vector pET-22b-*slcsn46s*. The expression vector pET-22b-*slcsn46s* was transformed to *E. coli* BL21 (DE3) to construct recombinant strain containing *slcsn46s*, which was confirmed by DNA sequencing. The recombinant strain containing slcsn46s was firstly cultivated in 10 mL LBA medium in 150 mL flasks and incubated at 37°C, 200 rpm until the OD_600_ reached 1.0. Then, 1 mL seed solutions were transferred into 100 mL LBA medium in 500 mL flasks and incubated at 37°C and 200 rpm. As the value of OD_600_ reached 0.6, 1 mM Isopropyl-beta-D-thiogalactopyranoside (IPTG) was added to culture solution and cultivated at 16°C and 200 rpm for 2 h. Finally, the recombinant strains was collected by centrifuging at 8,000 × g and 6°C for 10 min and disrupted by sonication. The method for purification of recombinant SlCsn46 was similar to previous studies (Qin et al., 2018b; Wang et al., 2021b) using Ni-IDA sefinose resin chromatography (Sangon Biotech, Shanghai, China). SDS-polyacrylamide gel electrophoresis (SDS-PAGE) was used to analysis the purified recombinant SlCsn46.

### Enzyme activity assay, substrate specificity, and kinetic parameters

The method for enzyme activity assay was same as previous studies (Wang et al., 2021a). Colloidal chitosan (0.5%, w/v) with 95% DA was used as substrate. To begin with, the diluted enzyme and substrate were preheating at 50°C for 2 min. After that, 50 μL diluted enzyme was added to substrate (350 μL) and incubated at 50°C for 10 min. Then, the reaction was stopped by addition with 600 μL 3,5-dinitrosalicylic acid (DNS) and the reaction solution were incubated at 100°C for 10 min. Finally, after centrifugation at 12,000 × g for 5 min, the absorbance of supernatant from reaction solution was measured at 540 nm. The reaction with DNS added before enzyme was used as controls. One unit of enzyme activity was defined as the amount of enzyme that releases 1 μmol reducing sugars per minute at 50°C.

The processes for determination of substrate specificity and kinetic parameters were same as previously described method (Wang et al., 2021a). For substrate specificity analysis, the relative activities against to different substrates, such as xylan, soluble starch, microcrystalline cellulose, colloidal and powder chitin, and colloidal chitosan with different DA (85–95%), were determined. For determination of kinetic parameters, different concentrations (1–10 mg/mL) of colloidal chitosan with 95% DA were used as substrate. The values of *K*_*m*_ and *V*_*max*_ were calculated by program Graft.

### Characterization of SlCsn46

The biochemical properties were determined according to the previous method (Wang et al., 2021a). The optimal pH of purified SlCsn46 was analyzed by detecting the relative activity at different pH in the range from pH 4.0 to 7.0. For pH stability of purified SlCsn46, the residual activity was detected after incubated at 25°C for 6 h in buffer with different pH (from pH 3.0 to 10.0). The residual activity of SlCsn46 treated with distilled water was set as 100%.

The temperature properties of purified SlCsn46 were composed of optimal temperature and thermal stability. For optimal temperature, the activities of purified SlCsn46 were detected at temperature from 30 to 70°C and the activity at 50°C was set as 100%. For thermal stability, the diluted purified SlCsn46 was incubated for 30 and 60 min at temperature from 40 to 65°C. The residual activity of purified SlCsn46 without heat treatment was set as 100%.

### Hydrolytic pattern of SlCsn46

The hydrolytic pattern of purified SlCsn46 was determined according to the already reported studies (Qin et al., 2018b; Guo et al., 2019; Luo et al., 2020) and the COSs with different DP (2 to 6) were used as substrate. Reactions were performed by addition with purified SlCsn46 to 0.5% (w/v) COSs and incubated at 50°C for different times (from 30 to 120 min). The composition of hydrolyzates from different COSs was detected by thin layer chromatography (TLC) method (Sun et al., 2020; Wang et al., 2021a).

### Efficient secreted expression of SlCsn46 in *Pichia pastoris* X33

The process for efficient secreted expression of SlCsn46 in *Pichia pastoris* X33 was similar to previous studies (Peng et al., 2013; Wang et al., 2019a). According to the preferred codons of *P. pastoris*, the sequences of slcsn46 without signal sequence (named *slcsn46opt*) was optimized, synthesized, and ligated to pGAPZαA by General Biotechnology (Chuzhou, China). The obtained expression vector pGAPZαA-*slcsn46opt* was linearized with *Bln*I and electrotransformed into *P. pastoris* X33. The method for isolation the recombinant strains was same as previous study (Wang et al., 2019b) and the detail protocol was provided in Supplementary material.

The recombinant strain with highest activity was cultivated in 7 L bioreactor. The method for high cell density fermentation was same as previous study (Wang et al., 2019b) and the detail protocol was provided in Supplementary material. The enzyme activity, total protein concentration, and wet cell weight were determined during the process of fermentation.

### Preparation of chitosan oligosaccharides

Preparation of COSs by purified SlCsn46 was similar to previous researches (Wang et al., 2021a). Different concentration (2% and 4%, w/v) of colloidal chitosan with 95% DA was used as substrate. Preparation of COSs was performed in 1,000 mL flask containing 200 mL colloidal chitosan addition with different amount of purified SlCsn46 (from 2 to 20 U/mL). The reactions were performed at 50°C and 100 rpm for 1 h, then, stopped by incubating at 85°C for 5 min. After centrifuging at 10,000 × g for 5 min, the supernatant containing COS were obtained, and used for subsequent tests.

Thin layer chromatography and high performance liquid chromatography (HPLC) were used to determine the composition of hydrolyzates. The method for HPLC was same to previous studies (Qin et al., 2018b; Wang et al., 2021a). Meanwhile, the hydrolysis rate was also detected based on the method described previously (Zhou et al., 2020).

### Preparation and chitosan oligosaccharides treatment of pre-packaged tofu

The fermented yellow whey tofu was manufactured according to Huang et al. (2021). Soybeans (300 g) were rinsed and soaked overnight in water at room temperature. Following this, they were drained and ground with water (ratio of dry beans to water = 1:8, w/w) in a blender (SJJ-20, Kangdeli Intelligent Technology Co., Ltd., Beijing, China). The mash then was micro-pressure boiled (105°C) for 6 min and filtered with a 75 μm screen. After the resulting soymilk cooled to approximately 80°C, 700 mL of the fermented yellow whey solution (coagulant) was slowly added into the soymilk (2,700 mL) under stirring slowly. The soymilk-coagulant suspensions were then held for 20 min to coagulate. The curds were broken, transferred into a cheesecloth-lined steel containers (11.1 cm × 7.1 cm × 5.5 cm) and pressed for 20 min using an automatic press machine. The tofu was then cut into small pieces (4.0 cm × 3.0 cm × 3.0 cm, 60 g), and immersed in sterilized water for 30 min to cool it.

Each tofu sample was placed in a single polyethylene container and randomly divided into four groups. Then, four groups of samples were added into each of the four solution (60 mL) at room temperature, including: (1) COSs-2–4 group (COSs with DP 2–4); (2) COSs-2–5 group (COSs with DP 2–5); (3) COSs-2–6 group (COSs with DP 2–6); (4) control group (sterile distilled water). The concentration of all COSs solutions were 4 mg/mL. All samples were pasteurized (85°C) for 30 min after packaging, and then refrigerated at 10°C for 15 days and analyzed every 3 days for microbial, physicochemical, and sensorial properties.

### Microbial analysis

Total viable count (TVC) was determined using the plate count agar method, as per the GB 4789.2–2016 of the Chinese standard. Briefly, 25 g of tofu samples were blended with 225 mL of sterile saline (0.85%). The mixture with appropriate dilutions was inoculated on plates and incubated at 36 ± 1°C for 48 h.

### Physicochemical analysis

For pH, tofu samples (25 g) were homogenized with 225 mL of distilled water. The pH value was measured by a pH meter (FE28-CN, Mettler Toledo, Zurich, Switzerland) at 25 ± 1°C.

The semimicro-quantitative nitrogent method was employed to determine the total volatile base nitrogen (TVB-N) of tofu, as per the GB 5009.228–2016 of the Chinese standard.

The water-holding capacity (WHC) of tofu was characterized using the method of Fan et al. (2021).

### Sensorial analysis

Sensory evaluation of tofu was performed by a sensory panel of twelve trained members (six female and six male). Panelists were requested to give a liking score for quality attributes like appearance, color, odor, and texture of the sample using a 9-point hedonic scale (9 was the highest quality score, 1 was the lowest and 5 was an acceptable level) (Laignier et al., 2022). Panelists were asked not to eat the samples, and the evaluation was made for only physical parameters.

### Statistical analysis

All the measurements were done in triplicate. The data were analyzed by the Statistical Package for the Social Sciences (SPSS) software (version 18.0, SPSS Inc., San Francisco, CA, United States) and are expressed as mean ± standard deviation (SD). Mean comparisons were performed by the Duncan’s Multiple Range Test (DMRT).

## Results and discussion

### Gene cloning and sequence analysis

**FIGURE 1:**
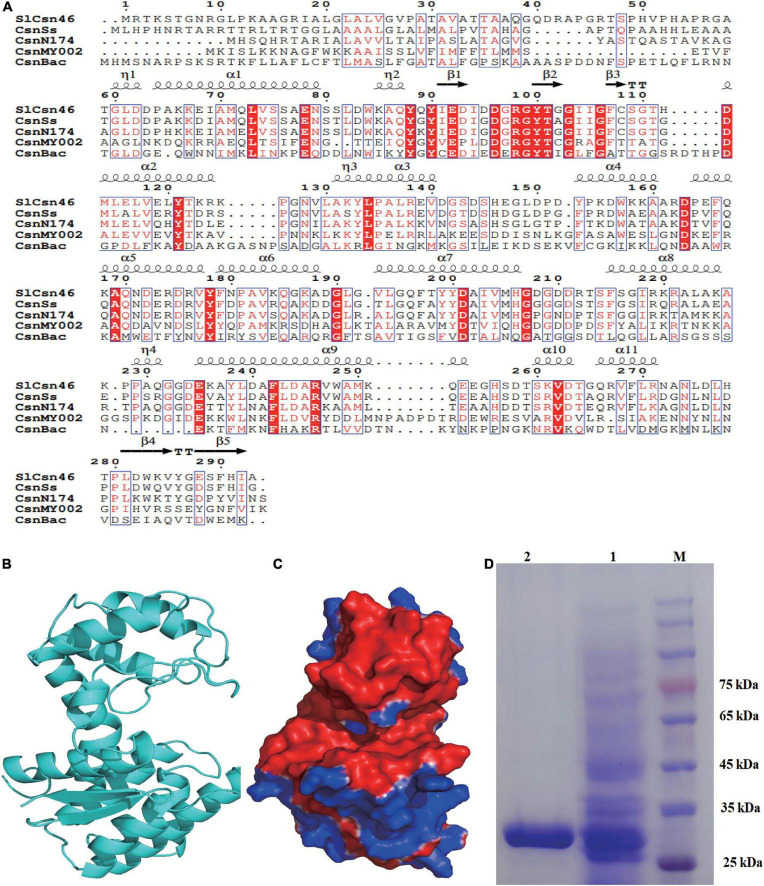
Sequence analysis and purification of SlCsn46. **(A)** Sequence alignment of SlCsn46 with already crystallized chitosanases. The listed sequences included the chitosanase CsnSs from *Streptomyces* sp. SirexAA-E (AEN13266.1), CsnN174 from *Streptomyces* sp. N174 (P33665.1), CsnMY002 from *Bacillus subtilis* (WP_148982050.1), and CsnBac from *Bacillus circulans* MH-K1 (P33673.2). **(B)** The overall structure of SlCsn46. **(C)** Surface electrostatic potential of SlCsn46. **(D)** SDS-PAGE analysis of the purified recombinant SlCsn46. 1: crude recombinant SlCsn46, 2: purified recombinant SlCsn46. M: protein marker.

After PCR amplification, a fragment about 900 bp was obtained and ligated to plasmid pMD20T. The result of DNA sequencing revealed that this fragment was 882 bp in length. The sequence identity of this fragment against different genes was performed by NCBIblastn. The results of NCBIblastn showed that the sequence of this fragment exhibited 96.5% identity to the hypothetical chitosanase from *S. lydicus* A02, followed by hypothetical chitosanase from *S. lydicus* 103 (83.2%) and *S. lydicus* M01 (82.9%). The obtained fragment was named as *slcsn46* and the open reading frame of *slcsn46* was 882 bp, which encodes 293 amino acids. The sequence of *slcsn46* was deposited on National Center of Biotechnology Information (NCBI) with accession number ON929883. The results of NCBIblastp analysis demonstrated that SlCsn46 was a chitosanase from GH46 family. Alignment SlCsn46 with already crystallized chitosanases indicated that Glu and Asp were catalytic residues (Figure 1A; Xu et al., 2022).

Based on the three-dimensional structure of chitosanase CsnN174 from *Streptomyces* sp. N174 [Protein Data Bank (PDB) deposition: 1chk.1], homology modeling was used to obtain the overall structure of SlCsn46. Similar to already crystallized chitosanases from GH46 family (Wang et al., 2020; Li et al., 2021; Xu et al., 2022), the three-dimensional structure of SlCsn46 is also α + β type, which is mainly composed of α-helices and β-strands. As shown in Figure 1B, the tertiary structure of SlCsn46 is dumbbell-shaped and consists of two globular domains, which is connected by long α-helices. Additionally, the results of vacuum electrostatic revealed that substrate binding region of SlCsn46 highly negatively charged, which is helpful to bind the cationic chitosan (Figure 1C).

### Purification, kinetic parameters, and substrate specificity

The recombinant SlCsn46 was expressed and purified from *E. coli* BL21 (DE3). The result of SDS-PAGE analysis showed that the purified recombinant SlCsn46 was about 30 kDa (Figure 1D). The specific activity of purified recombinant SlCsn46 was 1008.5 U/mg. The *K*_*m*_ and *V*_*max*_ of purified recombinant SlCsn46 were 1.92 mg/mL and 1,375.7 μM/min/mL, respectively. The substrate specificity of purified recombinant SlCsn46 was shown in Supplementary Table 1. The most favorable substrate for recombinant SlCsn46 was colloidal chitosan with 95% DA, followed by colloidal chitosan with 90% and 85% DA, respectively. Similar with many chitosanases from GH46 family (Sun et al., 2018, 2020; Wang et al., 2021a), purified recombinant SlCsn46 displayed no activity toward another polysaccharide such as chitin, xylan, microcrystalline cellulose, and soluble starch.

### Characterization of SlCsn46

**FIGURE 2:**
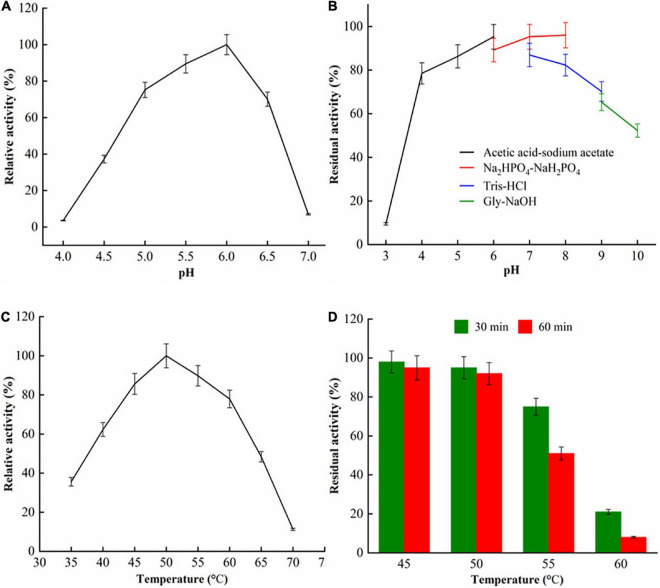
The characterization of purified SlCsn46. **(A)** Optimum pH, **(B)** pH stability, **(C)** optimum temperature, and **(D)** thermal stability.

The properties of pH and temperature of purified recombinant SlCsn46 were shown in Figure 2. For optimal pH, the purified recombinant SlCsn46 showed highest activity at pH 6.0 and retained above 70.3% relative activity in the range from pH 4.0 to 6.0 (Figure 2A). Generally, the optimal pH of chitosanases from GH46 family was in the range from 4.5 to 6.5. For instance, the optimal pH of chitosanases GsCsn46A, SaCsn46A, and BaCsn46B from *Gynuella sunshinyii*, *S. avermitilis*, and *Bacillus amyloliquefaciens* were 5.5, 6.2, and 6.5, respectively (Qin et al., 2018a; Luo et al., 2020; Guo et al., 2021). However, the chitosanases from *S. albolongus* ATCC27414 and *Bacillus* sp. MD-5 displayed maximum activity at pH 8.0 and 7.5, respectively (Guo et al., 2019; Yang G. et al., 2020). As shown in Figure 2B, the purified recombinant SlCsn46 exhibits excellent stability from pH 4.0 to 6.0 and the residual activity were all above 91.2%. According to the previous studies, we found that the pH stability of chitosanases from GH46 family varies greatly in accordance with their sources. The chitosanases from *S. avermitilis*, *B. mojavensis* SY1, and *S. hygroscopicus* R1 were stable from pH 4.0 to 8.0 (Guo et al., 2021; Wang et al., 2021a,b). Additionally, the residual activity of chitosanases from *Bacillus* sp. MD-5 decreased sharply as the treat pH above 7.0 (Yang G. et al., 2020). However, the recombinant chitosanases from *S. albolongus* ATCC 27414 and *S. niveus* exhibit excellent stability at higher pH (Guo et al., 2019; Chen et al., 2021). Generally, chitosanases display excellent properties at low pH is benefit for preparation of COSs since chitosan showed better solubility, which plays a pivotal role on the efficiency of decomposition of chitosan. In this study, SlCsn46 is active and stable from pH 4.5 to 6.0, which is suitable for preparation of COSs.

The purified recombinant SlCsn46 exhibited maximum activity at 50°C and remained active from 45 to 55°C (Figure 2C). Normally, the optimal temperature of chitosanases from GH46 family was in the range from 45 to 60°C. As depicted in Figure 2D, the purified recombinant SlCsn46 was stable when the incubating temperature below 50°C. As the incubating temperature increased to 55, 60, and 65°C, the residual activities were only 92.1, 75.3, and 23.2%, respectively. Based on the results from already reported studies, we found that the thermal stability of chitosanases from GH46 family varied greatly. For instance, the cold adaptive chitosanase from *G. sunshinyii* is only stable at the temperature from 5 to 35°C (Qin et al., 2018a). In addition, the chitosanases from *S. hygroscopicus* R1and *B. amyloliquefaciens* are mesophilic enzyme, which are stable at the temperature in the range from 35 to 55°C (Luo et al., 2020; Wang et al., 2021b). However, the thermal stable chitosanase from *Streptomyces roseolus* displays higher residual activity even though the incubating temperature reached 65°C (Jiang et al., 2012). In this study, the purified recombinant SlCsn46 was active and stable at 50°C demonstrating that the reaction temperature for preparation of COSs could set as 50°C which is helpful for reducing risk of microbial contamination.

### Effects of different metal ions on the stability of SlCsn46

For nine different metal ions, only Mn^2+^ could improve the stability and activity of SlCsn46. The residual activity of SlCsn46 treated with 1 and 5 mM Mn^2+^ were 121.3% and 163.5%, respectively (Supplementary Table 2). Metal ions Al^3+^ and Cu^2+^ displayed strong inhabitation on the stability of SlCsn46 and the residual activities of SlCsn46 treated with those two metal ions were below 9.3%. Metal ions, such as Co^2+^, Zn^2+^, Na^+^, Ca^2+^, K^+^, and Mg^2+^, showed little effect on the on the stability of SlCsn46 (Supplementary Table 2). Noticeably, metal ions are activator or inhibitor depends on the specific chitosanases. The result of this study and some previous researches (Viens et al., 2015; Cui et al., 2021) demonstrated that Cu^2+^ is an inhibitor. Otherwise, the chitosanase Sn1-CSN and Csn21c from *S. niveus* and *S. albolongus* ATCC 27414 are activated by Cu^2+^ and the activities of Sn1-CSN and Csn21c are up-regulated by 54.2 and 3%, respectively, when treated with 1 mM Cu^2+^ (Guo et al., 2019; Chen et al., 2021).

### Hydrolytic pattern of SlCsn46

**FIGURE 3:**
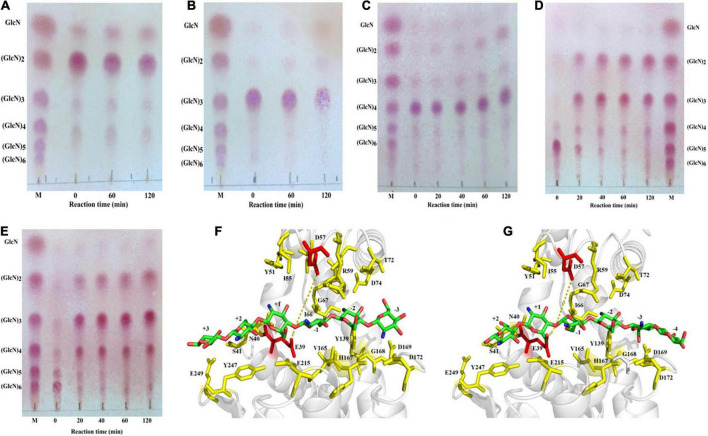
Analysis of the hydrolytic pattern of SlCsn46 toward different chitosan oligosaccharides (COSs). **(A)** Chitobiose, **(B)** chitotriose, **(C)** chitotetraose, **(D)** chitopentaose, **(E)** chitohexaose. **(F)** The “3 + 3” splitting mode of SlCsn46 toward (GlcN)_6_. **(G)** The “4 + 2” splitting mode of SlCsn46 toward (GlcN)_6_. The amino acids residues E39 and D57 colored in red are catalytic active sites. M: Chitosan oligosaccharides marker.

The purified recombinant SlCsn46 exhibited no activity toward COSs with DP below 4 (Figures 3A,B). The (GlcN)_2_ and (GlcN)_3_ were not decompose and transformed to smaller products even though the reaction time reached 120 min. In addition, as DP of COSs was larger than 3, the hydrolysis efficiency of purified recombinant SlCsn46 increased quickly (Figures 3C–E). As shown in Figure 3C, few (GlcN)_4_ was decomposed and converted to (GlcN)_2_ revealing that the smallest substrate of SlCsn46 was (GlcN)_4_. Different from (GlcN)_4_, (GlcN)_5_, and (GlcN)_6_ were completely cleaved and transformed to smaller COSs with DP 2 to 4 as the reaction time reached 40 and 20 min, respectively (Figures 3D,E). Furthermore, no GlcN and COSs with higher DP than the corresponding substrates were detected during the process of hydrolysis demonstrating that SlCsn46 is an endo-type chitosanase without transglycosylation activity.

According to the results of hydrolytic pattern of SlCsn46 and previous studies (Wang et al., 2020; Li et al., 2021), we deduced that there are two binding and cutting modes for SlCsn46 interacted with substrates. For instance, the products of (GlcN)_6_ were composed of (GlcN)_2_, (GlcN)_3_, and (GlcN)_4_, which are involved with “4 + 2” and “3 + 3” splitting mode, respectively. For “3 + 3” splitting mode, the (GlcN)_6_ enters into the active region of SlCsn46 and the six sugar units of (GlcN)_6_ are located at the –3 to + 3 subsites (Figure 3F). Then, several key amino acids (E39, N40, S41, Y51, I55, D57, R59, I66, G67, T72, D74, Y139, V165, H167, G168, D169, D172, E215, Y247, and E249) related to recognition and catalysis of (GlcN)_6_ are formed some hydrogen bonds with the amino and group hydroxyl of (GlcN)_6_. For example, the amino group at the C^–2^ position of +1 subsite may form two hydrogen bonds with the side chain of E39 and Y51. The hydroxyl group at the C^–6^ position of +2 subsite may form three hydrogen bonds with the side chain of E39, N40, and S41 (Figure 3F). For “4 + 2” splitting mode, the (GlcN)_6_ enters into the active region of SlCsn46 and the six sugar units are located at the –4 to +2 subsites (Figure 3G).

### Efficient secreted expression of SlCsn46 in *Pichia pastoris* X33

The biochemical properties of recombinant SlCsn46 exhibited its potential application value for preparation of COSs with different DP. However, heterologous expression of SlCsn46 in *E. coli* BL21 limited its further industrial application on preparation of COSs. Thus, it is necessary to develop an effective method for efficient secreted expression of SlCsn46. A series of recently reported researches demonstrated that *P. pastoris* is a suitable host for heterologous production of recombinant chitosanases due to its high expression level, extracellular secretion as well as mature fermentation process (Ahmad et al., 2014). Nevertheless, methanol-inducible is the most preferred way to prepare recombinant chitosanases for already report studies. Compared with methanol-inducible, constitutive expression of recombinant protein in *P. pastoris* has several advantages such as simplifies cultivation efforts, omits the use of methanol as inducer and shortens fermentation time (Looser et al., 2015). Therefore, constitutive expression is an alternative way to prepare recombinant SlCsn46.

**FIGURE 4:**
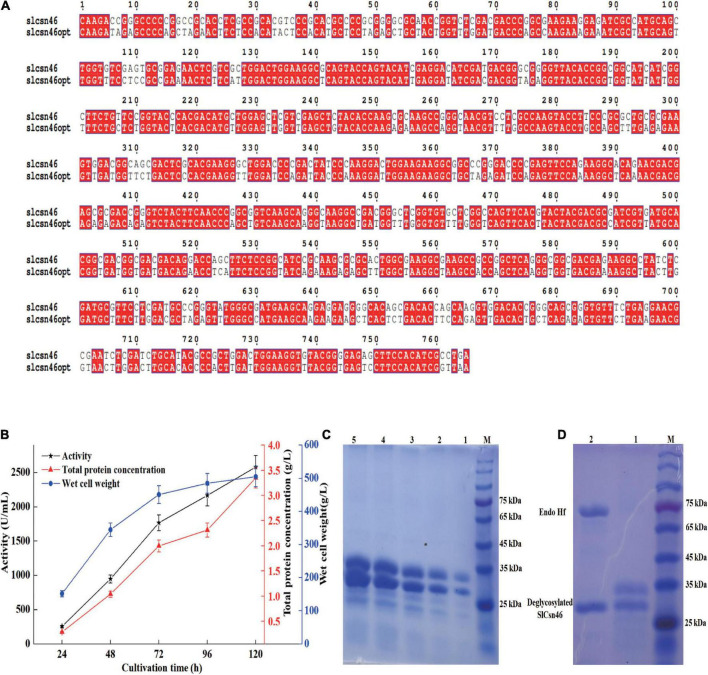
Efficient secreted expression of SlCsn46 in *Pichia pastoris* X33. **(A)** Sequence comparison between the native and optimized gene. **(B)** The chitosanase activity, total protein concentration, and wet cell weight during fed-batch fermentation in 7 L bioreactor. **(C)** SDS-PAGE analysis of supernatants from different cultivation time. M represents marker, lanes 1–5 represent supernatant from 24 to 120 h. **(D)** SDS-PAGE of SlCsn46 after Endo Hf treatment. M represents marker, lanes 1 and 2 represents supernatant from 48 h and de-glycosylated SlCsn46.

The codon usage analysis indicated that the codon usage between *P. pastoris* and *S. lydicus* varied greatly. Therefore, the GC content and codons rarely used in *P. pastoris* were optimized based on the preference of *P. pastoris* (Figure 4A). The optimized gene *slcsn46opt* was ligated to constitutive vector pPGAPZαA and transformed to *P. pastoris* X33. A recombinant strain (named X33-15) with the highest activity was isolated and cultivated in 7 L bioreactor. As shown in Figure 4B, the maximum activity and total protein concentration of X33-15 were 2651 U/mL and 3.35 g/L, respectively. SDS-PAGE analysis found that the supernatants from different induction time were mainly composed of recombinant SlCsn46 (Figure 4C). In addition, there were two bands on SDS-PAGE, which were about 34 and 26 kDa, respectively (Figure 4C). The band with about 34 kDa may probably due to glycosylation. N-glycosylation site analysis exhibited SlCsn46 has one potential N-glycosylation site (N^79^SSL). As depicted in Figure 4D, there was only one band about 26 kDa after de-glycosylation revealing that the band with 34 kDa was a glycoprotein (Figure 4D).

### Controllably preparation of chitosan oligosaccharides with desirable degree of polymerization by SlCsn46

Recently, a series of previous studies indicated that the bioactivity of COS is related to its DP. For instance, Zhao et al. (2022) found that the inhibitory effect on *Salmonella* of (GlcN)_4_ is about 2-fold higher than (GlcN)_2_. Similarly, a previous study revealed that the antimicrobial activity against *Staphylococcus aureus* is also related to DP and the COS with DP below than five showed no antibacterial activity (Li et al., 2014). Thus, it is necessary to prepare COSs with desirable DP according to its subsequent industrial application.

**FIGURE 5:**
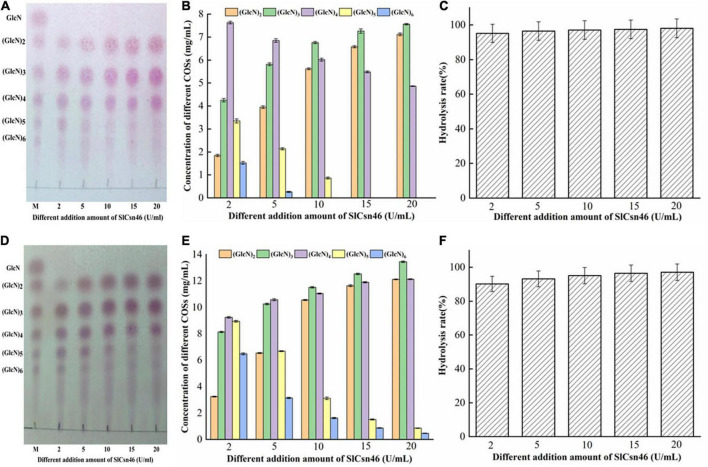
Controllable preparation of chitosan oligosaccharides (COSs) by SlCsn46. **(A)** Thin layer chromatography (TLC) analysis of hydrolyzates from 2% (w/v) colloidal chitosan addition with different amounts of SlCsn46 (2–20 U/mL). **(B)** High performance liquid chromatography (HPLC) analysis of COSs output from 2% colloidal chitosan addition with different amounts of SlCsn46 (2–20 U/mL). **(C)** The hydrolysis rate of 2% colloidal chitosan addition with different amounts of SlCsn46 (2–20 U/mL). **(D)** TLC analysis of hydrolyzates from 4% (w/v) colloidal chitosan addition with different amounts of SlCsn46 (2–20 U/mL). **(E)** HPLC analysis of COSs output from 4% colloidal chitosan addition with different amounts of SlCsn46 (2–20 U/mL). **(F)** The hydrolysis rate of 4% colloidal chitosan addition with different amounts of SlCsn46 (2–20 U/mL). M: Chitosan oligosaccharides marker.

The different concentration (2% and 4%, w/v) of colloidal chitosan with 95% DA addition with different amounts of recombinant SlCsn46 (2–20 U/mL) were used to prepare COSs with desirable DP. The results of 2% colloidal chitosan added with 2–20 U/mL SlCsn46 were shown in Figures 5A–C. The results of TLC revealed that the hydrolysis of 2% colloidal chitosan were mainly composed of (GlcN)_3_ to (GlcN)_5_ when the amount of purified recombinant SlCsn46 was 2 U/mL (Figure 5A). As the amounts of SlCsn46 above 5 U/mL, the final products mainly included (GlcN)_2_ to (GlcN)_4_ (Figure 5A). Meanwhile, the concentrations of different COSs were determined by HPLC method. For the amount of SlCsn46 was 2 U/mL, the concentrations of (GlcN)_3_, (GlcN)_4_, and (GlcN)_5_ were 4.25, 7.62, and 3.35 mg/mL, respectively, which dominated 81.9% of the total COSs (Figure 5B). Furthermore, the hydrolysis rates of all reactions were above 95.2% (Figure 5C). As the substrate changed to 4% (w/v) colloidal chitosan, the end products of hydrolyzates exhibited some difference (Figures 5D–F). For 2 U/mL, the end products were mainly composed of (GlcN)_2_ to (GlcN)_6_ and the concentrations of those COSs were 3.25, 8.15, 9.25, 8.95, and 6.48 g/L, respectively (Figures 5E,F). For 5 U/mL, the hydrolyzates mainly included (GlcN)_2_ to (GlcN)_5_. As the amounts of SlCsn46 above 5 U/mL, the final products mainly included (GlcN)_2_ to (GlcN)_4_ (Figures 5E,F). In addition, the total COSs yields of all reactions were also above 90.2% (Figure 5F).

Recently, several chitosanases from different GH families, such as GH8, GH46, and GH75 family, have been used to prepare COSs (Zhou et al., 2020; Jiang et al., 2021; Wang et al., 2021a). Among those studies, chitosanases from GH46 family display higher efficiency than that from GH8 and GH75 family. For instance, the chitosanase CsnBm from *B. mojavensis* SY1 could decompose 91.2% of colloidal chitosan (4%, w/v) into COSs with DP 2–6 after 1 h reaction in the presence of 5 U/mL of CsnBm (Wang et al., 2021a). Similarly, the chitosanases ShCsn46 and BaCsn46A from *S. hygroscopicus* R1 and *B. amyloliquefaciens* also exhibit high efficiency toward 4% and 3% colloidal chitosan (Wang et al., 2021b). However, a chitosanase (named PbCsn8) from *Paenibacillus barengoltzii* belonged to GH8 family shows lower activity against 5% colloidal chitosan and the hydrolysis rate is 79.3% after 4 h reaction in the presence of 5 U/mL of PbCsn8 (Jiang et al., 2021). Additionally, the hydrolysis rate of the chitosanase Csn75 from GH75 family is 90.65% when using 2% colloidal chitosan addition with 30 U/mL of Csn75 (Zhou et al., 2020). In this study, the hydrolysis rates of all reactions were above 90.1% regardless the concentration of substrate and the amounts of SlCsn46. Furthermore, COSs with DP 2–4, 2–5, and 2–6 were controllably prepared by adjusting the addition of SlCsn46. The above results demonstrated that SlCsn46 with great potential and competitiveness for preparation of COSs mixture with desirable DP.

### Potentionl application of chitosan oligosaccharides in preservation of pre-packaged tofu

Food products have different characteristics that can affect the efficiency of antimicrobial inhibitors. To further confirm the antibacterial activity of different hydrolysis product prepared by SlCsn46, COSs with DP 2–4, 2–5, and 2–6 were added into the pre-packed tofu, and the changes in quality attributes of tofu during the storage was monitored.

### Microbiological analysis

**FIGURE 6:**
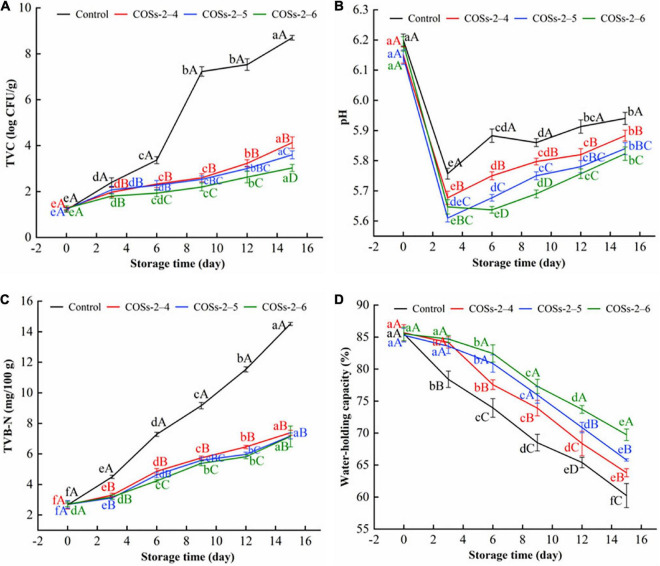
Effect of chitosan oligosaccharides (COSs) on total viable count (TVC) **(A)**, pH **(B)**, total volatile base nitrogen (TVB-N) **(C)**, water-holding capacity (WHC) **(D)** of pre-packaged tofu during storage of 15 days at 10°C. Control: sterile distilled water; COSs-2–4, COSs-2–5, and COSs-2–6: pre-packaged tofu added with 4 mg/mL COS with DP 2–4, COS with DP 2–5, COS with DP 2–6, respectively. Different lowercase letters indicate significant differences in the same group of samples at different storage time (*P* < 0.05). Different capital letters indicate significant differences in the different group of samples within the same storage time (*P* < 0.05).

Total viable count of tofu with different COSs treatments in comparison with the control group during the storage of 15 day at 10°C are showed in Figure 6A. The TVC was increased in all groups with the extension of the storage time, of which the TVC of COSs added sample were all dramatically lower than that of the control group (*P* < 0.05). The results indicated that COSs could inhibit the growth of microorganism in tofu. Numerous studies have shown that the antibacterial activity of COSs is largely associated with the interaction between its positively charged (${-\text{NH}}_{3}^{+}$) groups and the negatively charged components (lipopolysaccharides, phospholipids, or lipoproteins) of the inner and outer membranes of the microbial cell membrane (Ke et al., 2022). The interaction could deforme mycelia and change the permeability of cell walls and membranes, and release the intracellular substances (Meng et al., 2022). Meanwhile, COSs can infiltrate into the nucleus *via* the reduction of chitin production and the enhancement of chitinase activity. Those phenomena disrupted normal cellular metabolism and eventually inhibited the growth of food-spoilage microorganisms (Zhao et al., 2022). Singh and Benjakul (2020) also found Asian sea bass slices added with COSs at various concentrations had lower bacterial counts than controls during storage at 4°C. Similar results were also obtained in yellowfin tuna slices added with 0.2 and 0.4 mg/g of COSs (Singh et al., 2021b). Of note, COSs-2–6 groups had lower TVCs than COSs-2–4 and COSs-2–5 groups at the whole storage period. The excellent antimicrobial activity of COSs monomer with higher DP has been documented (Zhao et al., 2022).

### pH analysis

The change of the pH value on tofu samples during the storage of 15 day at 10°C are presented in Figure 6B. At day 3, pH value of tofu without and with treatments decreased dramatically. This was possibly because lactic acid bacteria as the main spoilage bacteria in tofu continue to grow and produce organic acids during earlier period (Rossi et al., 2016). But, Kim et al. (2004) found that no significant pH change in chitosan tofu before 10 days of storage at 4°C. Moreover, the pH value of the COSs treatment groups was significantly lower than that of the control group, which was caused by the presence of acetic acid in the COSs solution. During 3–15 days of storage, pH values enhanced in all groups to some extent, and the values of the control group were always higher than those of the COSs treatment groups (*P* < 0.05). The findings were in agreement with those of Chen et al. (2014), who reported that the pH change of Lactic acid bacteria tofu was closely related to storage time. Meanwhile, Chen et al. (2014) found that microbial metabolism in tofu was the main contributors responsible for the variations in pH. Besides, Singh et al. (2021a) noted that the inhibition of COSs treatment on lipid oxidation was one of important cause for the smaller change in pH.

### Total volatile base nitrogen analysis

Total volatile base nitrogen, a vital index of food spoilage, is mainly attributed to the influence of the spoilage microorganisms (Zhao et al., 2022). In Figure 6C, prior to the 3rd day, the TVB-N values were enhanced slowly with no significant difference of values among COSs treated groups. During 6–15 days of storage, TVB-N of Control group enhanced sharply from 7.59 mg/100 g to final value of 14.51 mg/100 g. Compared with the control group, the significant reductions (*P* < 0.05) of almost 31.4–50.8% were observed in COSs treatment groups. Jia et al. (2018) also reported the dramatic reduction of TVB-N value in silver carp after treated 1% (w/v) COS. Additionally, a similar trends of TVB-N existed in carp filets from control or COSs treated groups (Yu et al., 2021). The lower TVB-N content in treated groups was primarily attributed to the antimicrobial capacity of COSs, since microbial activity is the main cause for TVB-N accumulation of protein-rich food (No et al., 2007).

### Water-holding capacity analysis

Water is one of the main components of tofu, and plays a key role in maintaining quality stability and structure of tofu (Lu et al., 2022). As an effective quality indicator, WHC from each group showed similar trend with decrease during storage (Figure 6D). Previous literature stated that WHC is attributed to the contribution of strength of tofu gel networks (Fan et al., 2021). The decrease of WHC in each groups means the decline of the ability of tofu gel matrices to immobilize water molecules. In addition, the significant decrease of WHC in the control group during storage was still observed. Ye et al. (2021) also found that the water loss caused textural deterioration of tofu gels. The sensory results in this manuscript (Table 1) confirmed this to some extent.

**TABLE 1:** Scores of sensory evaluation on pre-packaged tofu added with chitosan oligosaccharides (COSs) in the storage period.

Sensory evaluation	Groups	Storage time (days)					
		0	3	6	9	12	15
Appearance	Control	8.47 ± 0.15^aA^	8.23 ± 0.21^aA^	7.40 ± 0.36^bB^	6.67 ± 0.25^cB^	4.87 ± 0.21^dB^	3.67 ± 0.15^eC^
	COSs-2–4	8.37 ± 0.12^aA^	8.27 ± 0.06^aA^	7.70 ± 0.26^bAB^	7.47 ± 0.15^cCA^	7.20 ± 0.17^cA^	6.80 ± 0.10^dB^
	COSs-2–5	8.43 ± 0.21^aA^	8.27 ± 0.15^aA^	7.80 ± 0.10^bAB^	7.57 ± 0.21^cA^	7.23 ± 0.15^cA^	6.97 ± 0.21^cAB^
	COSs-2–6	8.40 ± 0.20^aA^	8.27 ± 0.15^aA^	7.93 ± 0.06^bA^	7.50 ± 0.10^cA^	7.30 ± 0.26^cdA^	7.13 ± 0.12^dA^
Color	Control	8.53 ± 0.21^aA^	7.63 ± 0.23^bAB^	7.20 ± 0.30^cC^	7.17 ± 0.15^cC^	6.77 ± 0.15^deB^	6.43 ± 0.31^eB^
	COSs-2–4	8.50 ± 0.10^aA^	7.83 ± 0.15^bC^	7.50 ± 0.20^cBC^	7.37 ± 0.06^cdBC^	7.13 ± 0.21^dAB^	6.80 ± 0.21^eAB^
	COSs-2–5	8.50 ± 0.20^aA^	7.93 ± 0.15^bAB^	7.80 ± 0.17^bcAB^	7.53 ± 0.15^cdAB^	7.23 ± 0.31^deA^	6.93 ± 0.12^eA^
	COSs-2–6	8.53 ± 0.06^aA^	8.13 ± 0.21^bA^	8.00 ± 0.10^bcA^	7.77 ± 0.21^cA^	7.43 ± 0.12^dA^	7.17 ± 0.15^dA^
	Control	8.40 ± 0.20^aA^	8.10 ± 0.26^aA^	6.43 ± 0.35^bC^	4.43 ± 0.15^cC^	2.07 ± 0.21^dC^	1.33 ± 0.25^eC^
Odor	COSs-2–4	8.43 ± 0.15^aA^	8.20 ± 0.20^aA^	7.40 ± 0.10^bB^	7.17 ± 0.06^bB^	6.67 ± 0.15^cB^	6.03 ± 0.21^dB^
	COSs-2–5	8.33 ± 0.06^aA^	8.13 ± 0.15^aA^	7.57 ± 0.15^bAB^	7.07 ± 0.15^cB^	6.80 ± 0.10^dAB^	6.27 ± 0.21^eB^
	COSs-2–6	8.43 ± 0.21^aA^	8.17 ± 0.12^aA^	7.90 ± 0.10^bA^	7.43 ± 0.15^cA^	7.07 ± 0.12^dA^	6.70 ± 0.20^eA^
	Control	8.43 ± 0.15^aA^	8.17 ± 0.12^aA^	7.57 ± 0.21^bB^	6.03 ± 0.25^cB^	4.73 ± 0.06^dB^	4.27 ± 0.35^eC^
Texture	COSs-2–4	8.40 ± 0.22^aA^	8.17 ± 0.21^aA^	7.67 ± 0.15^bB^	7.37 ± 0.06^bcA^	7.10 ± 0.10^cA^	6.33 ± 0.31^dB^
	COSs-2–5	8.47 ± 0.15^aA^	8.30 ± 0.20^aA^	7.87 ± 0.15^bAB^	7.57 ± 0.06^cA^	7.27 ± 0.12^dA^	6.73 ± 0.25^eAB^
	COSs-2–6	8.47 ± 0.12^aA^	8.23 ± 0.15^aA^	8.00 ± 0.10^bcA^	7.63 ± 0.21^cdA^	7.27 ± 0.32^deA^	7.00 ± 0.26^eA^

In the same column, the significant difference was marked as different lowercase letters (*P* < 0.05); similarly, in the same row, the significant difference was marked as different capital letters (*P* < 0.05).

### Sensory analysis

As shown in Table 1, on day 0, no difference of appearance, color, odor, and texture likeness scores were observed in tofu samples without and with COSs treatment (*P* > 0.05), indicating that no negative effect of COSs addition on the sensory quality of tofu. Thereafter, sensory scores of all parameters for each group were decreased with storage time extension. Among them, the odor scores of control dramatically declined from the 9th day (*P* < 0.05), and were all lower than the threshold of sensory unacceptability, which was basically consistent with the results of TVC evaluation. In contrast, the scores for all sensory attributes in COSs treated groups were always higher than five (acceptable score) throughout the storage period, indicating that COSs with DP 2–4, 2–5, and 2–6 all have the potential to extend the shelf life of tofu. Higher sensory scores in COSs treatment group were more likely due to the lower TVB content, which resulted in a reduction in off-flavor and offensive odor production (Figure 5C). Among groups, COSs-2–6 treated groups had the smallest decline rate of appearance, color, odor, and texture score with the final value reaching 7.13, 7.17, 6.70, and 7.00, respectively. Apparently, COSs-2–6 addition could enhance the shelf-life of tofu while maintaining better sensory quality when compared to COSs-2–4 and COSs-2–5 addition.

Based on microbial, physicochemical, and sensorial evaluation above, the shelf life of pre-packaged tofu was nearly 6 days for control, and 15 days for all COSs treatment group.

## Conclusion

In conclusion, a chitosanase (SlCsn46) from *S. lydicus* S1 was bioinformatics analyzed, purified, and characterized. SlCsn46 belonged to GH46 family and the specific activity of purified SlCsn46 was 1008.5 U/mg. The purified SlCsn46 exhibited maximum activity at pH 6.0 and 50°C. Additionally, Mn^2+^ could as an activator to improve the stability of SlCsn46. Meanwhile, SlCsn46 is an endo-type chitosanase and exhibited high efficiency toward 2% and 4% colloidal chitosan to produce COSs with desirable DP. Furthermore, COSs with DP 2–4, 2–5, and 2–6 effectively prolonged the shelf-life of pre-packed tofu compared to the control. The results of this study indicating that SlCsn46 is suitable for controllable preparation of COSs with desired DP and the COSs exhibit great potential value for preservation of pre-packaged tofu.

## Data availability statement

The datasets presented in this study can be found in online repositories. The names of the repository/repositories and accession number(s) can be found below: NCBI with accession number: ON929883.

## Author contributions

HC contributed to gene clone, construct recombinant strain, and bioinformatics analysis of SlCsn46 and writing. BL contributed to potentionl application of COSs in preservation of pre-packaged tofu. RZ contributed to characterization, purification, and kinetic parameters of SlCsn46. ZG contributed to preparation and COSs treatment of pre-packaged tofu. MW contributed to hydrolytic pattern of SlCsn46. JZ contributed to large scale production of SlCsn46. WS contributed to project administration. LZ contributed to funding acquisition. JW contributed to gene cloning, signal peptide optimization, efficient secreted expression, and experiments planning. All authors contributed to the article and approved the submitted version.
