# Supplementary material for: Controllable preparation of chitosan oligosaccharides via a recombinant chitosanase from marine Streptomyces lydicus S1 and its potential application on preservation of pre-packaged tofu
Source: Front Microbiol. 2022 Sep 26;13:1007201. doi: 10.3389/fmicb.2022.1007201 (PMC9549211; doi:10.3389/fmicb.2022.1007201)
Supplement: Supplementary file 1 [file Data_Sheet_1.docx]

Supplementary Material

**Supplementary Methods**

**Protocol for Selection of Recombinant Strains**

The transformants were picked and cultured in 24-deep-well microplates containing 1.8 mL/well BMDY medium at 30 ºC. After 36 h, plates were subjected to centrifugation and supernatants were used in subsequent activity assays. The clones showing higher activities were checked by shaking flask fermentation.

**Shake Flask Cultures**

The single-copied recombinant strains were inoculated into 10 mL BMDY medium in a 150 mL flask and incubated at 30 °C and 200 rpm for 48 h. Then 1 mL culture was harvested for chitosanase assay.

**Protocol for High Cell Density Fermentation**

Inoculum was cultured in BMGY medium. Cells were grown for 18–20 h at 30 °C on shaker of 200 rpm. Then, 10% (v/v) of the inoculum was inoculated into the 7 L bioreactors containing 2.5 L basal salt medium, which made of 0.47 g/L CaSO_4_·2H_2_O, 9.1 g/L K_2_SO_4_, 7.5 g/L MgSO_4_·7H_2_O, 6.2 g/L KOH, 13.35 mL/L H_3_PO_4_ (85%), 20.0 g/L glycerol and 1.5 mL Pichia trace metal 1 (PTM1). One liter PTM1 consists of 6 g CuSO_4_·5H_2_O, 0.08 g NaI, 3 g MnSO_4_·H_2_O, 0.5 g CoCl_2_, 20 g ZnCl_2_, 0.02 g H_3_BO_3_, 0.2 g Na_2_MnO_4_·2H_2_O, 65 g FeSO_4_·7H_2_O, 0.2 g biotin and 30 mL (NH_4_)_2_SO_4_. The temperature was controlled at 30 °C and the pH was maintained at 5.0 using NH_4_OH (28%) and H_3_PO_4_ (10%). The agitation rate was set at 700 rpm and the aeration rate was 7.5 L/min. The recombinant strains were cultivated on BSM medium and using glycerol as sole carbon source. The concentration of glycerol was kept stable by monitoring the DO content and maintaining it at greater than 10%. The enzyme activity, total protein concentration and dry cell weight were monitored throughout the cultivation.

**Supplementary results**

**Supplement Table S1** The substrate specificity of SlCsn46

| Substrates | Residual activity (%) |
| --- | --- |
| Colloidal chitosan with 85% DA | 90.5 |
| Colloidal chitosan with 90% DA | 95.3 |
| Colloidal chitosan with 95% DA | 100 |
| Microcrystalline cellulose | ND* |
| Colloidal chitin | ND |
| Powder chitin | ND |
| Soluble starch | ND |
| Xylan | ND |

ND* represents the enzyme activity was not detected.

**Supplement Table S2** Effects of different metal cations on SlCsn46 stability

| Metal ions | Residual activity (%) | |
| --- | --- | --- |
|  | 1 mM | 5 mM |
| Na^+^ | 92.3 | 89.1 |
| K^+^ | 93.1 | 94.5 |
| Cu^2+^ | 9.3 | 5.6 |
| Mn^2+^ | 121.3 | 163.5 |
| Mg^2+^ | 89.3 | 86.5 |
| Co^2+^ | 89.6 | 88.5 |
| Al^3+^ | 8.6 | 4.6 |
| Zn^2+^ | 80.6 | 82.3 |
| Ca^2+^ | 95.3 | 92.1 |
